# Clinical significance of endometrial abnormalities: an observational study on 1020 women undergoing hysteroscopic surgery

**DOI:** 10.1186/s12905-022-01682-5

**Published:** 2022-04-07

**Authors:** Lodovico Patrizi, Carlo Ticconi, Barbara Borelli, Susanna Finocchiaro, Carlo Chiaramonte, Francesco Sesti, Alessandro Mauriello, Caterina Exacoustos, Luisa Casadei

**Affiliations:** 1 Department of Surgical Sciences, Section of Gynecology and Obstetrics, University of Rome Tor Vergata, Rome, Italy; 2 Department of Experimental Medicine, University of Rome Tor Vergata, Rome, Italy; 3 Department of Biomedicine and Prevention, University Tor Vergata, Rome, Italy

**Keywords:** Endometrial atypia and cancer, Endometrial polyps, AUB, Menopause, Transvaginal ultrasonography, Hysteroscopy

## Abstract

**Background:**

The overall clinical significance of the finding of endometrial abnormalities in predicting premalignant/malignant endometrial lesions is still incompletely determined. For this reason the management, surgical or expectant, of women in which an endometrial abnormality has been detected is debated.

**Methods:**

This retrospective study was carried out on 1020 consecutive women, 403 premenopausal and 617 postmenopausal, who underwent operative hysteroscopy in a University Hospital for suspected endometrial abnormalities, which were detected by transvaginal ultrasound (TVS) and/or office hysteroscopy. In these women, the clinical characteristics and findings at TVS and hysteroscopy were evaluated in relation to the presence/absence of premalignant/malignant endometrial lesions at pathology report.

**Results:**

The clinical characteristics considered were significantly different when the study women were compared according to their menopausal status. Premalignant/malignant lesions were found in 34/1020 (3.33%) women. Complex hyperplasia with atypia and endometrial cancer were detected in 22 (2.15%) and 12 (1.17%) cases, respectively. The postmenopausal women had a significantly higher risk of premalignant/malignant lesions than premenopausal women (O.R. = 5.098 [95% C.I.: 1.782–14.582], *P* < 0.005). This risk was even higher when abnormal uterine bleeding (AUB) was present (O.R. = 5.20 [95% C.I.: 2.38–11.35], *P* < 0.0001). The most significant associations with premalignant/malignant endometrial lesions were BMI, AUB in postmenopause, overall polyp size, atypical aspect of endometrial polyps at hysteroscopy, postmenopausal status, diabetes mellitus and patient age.

**Conclusions:**

The results of the present study suggest that the proper, aggressive or expectant, management of endometrial abnormalities should take into account both ultrasonographic and hysteroscopic findings together with the specific clinical characteristics of the patients.

## Introduction

Many studies have been carried out to establish the premalignant/malignant potential of specific endometrial abnormalities, such as polyps [1–5], thickened endometrium [6, 7] or alterations of the endometrial stripe that are detected by imaging in women with or without abnormal uterine bleeding (AUB) [8, 9]. Management guidelines have been proposed accordingly [10–13] and risk factors for premalignant/malignant lesions of the endometrium have been established [14–16]. However, relatively less experimental information is available on the overall clinical significance of the finding of an endometrial abnormality observed at imaging before the final histologic diagnosis.

Endometrial abnormalities are frequently found in women in their late reproductive age, in menopause and in post-menopause. These abnormalities can be found during a transvaginal sonography (TVS) in case of specific symptoms such as AUB or pelvic pain, but also in asymptomatic women who have undergone some imaging (magnetic resonance, computerized tomography, abdominal sonography) for non-gynecologic symptoms or who had an office TVS during a routinary gynecologic check. They are due to a variety of underlying dysfunctional, benign, premalignant and malignant endometrial conditions often coexisting in the same patient. Moreover, they can also be associated with other concomitant problems not directly related to the endometrium, such as hypertension, obesity or tamoxifen treatment for estrogen receptor-positive (ER+) breast cancer. A complete diagnostic workup such as an office hysteroscopy cannot be carried out for all women due to several reasons including excessive patient discomfort, stenosis of the cervical uterine os, presence of concomitant other conditions such as heart disease, or excessive vaginal bleeding; these barriers can prevent the procedure and do not allow to obtain a final histological result unless operative hysteroscopy is carried out. For all these reasons, the finding of endometrial abnormalities can still represent a challenge for clinicians, mainly with regard to the prediction of benign, premalignant or malignant endometrial lesions.

In order to further clarify the overall clinical significance of the finding of endometrial abnormalities and to further improve the successive management of patients, the present study was carried out to investigate the association between several clinical conditions and risk factors for premalignant/malignant endometrial lesions in a population of women consecutively referred for hysteroscopic removal of endometrial lesions. Specific attention was paid to the association between the menopausal status of the study subjects and the risk of premalignant/malignant endometrial histology, since there is clear evidence that this risk is increased in postmenopausal women [17, 18].

## Methods

### Subjects and study design

This observational retrospective study included 1020 consecutive women who underwent operative hysteroscopy at Policlinico Tor Vergata University Hospital, Rome, Section of Gynecology, between January 1st, 2014 and September 30th, 2020. The study was carried out in accordance with the Helsinki Declaration for Medical Research involving Human Subjects and was approved by the Institutional Review Board of Policlinico Tor Vergata University Hospital (Protocol number: 110/19).

Preoperative diagnosis has been made by TVS followed, whenever possible, by diagnostic office hysteroscopy. The indications for TVS were: (a) scheduled check in women treated with tamoxifen for ER+ breast cancer; (b) pelvic pain of unknown origin; (c) abnormal uterine bleeding (AUB). Moreover, abnormal endometrial findings were detected in several asymptomatic women (no AUB, no pelvic pain, no gynecologic symptoms) who underwent either office US during a routinary gynecologic visit as an extension of physical examination of the patient or in women undergoing other imaging techniques different from TVS for the diagnosis of non-gynecologic diseases. These women were then referred to our center for further investigation and/or surgery. The indication for office hysteroscopy was any abnormal endometrial finding at TVS, irrespective of the symptomatic/asymptomatic status of the women.

Indications for operative hysteroscopy were endometrial abnormalities detected by TVS and/or hysteroscopy. As a general rule at our institute, all the patients scheduled for surgery during the pre-hospitalization procedure underwent an accurate anamnestic general and gynecologic investigation, a pelvic examination, an internal TVS check before intervention, carried out following the IETA criteria to describe the sonographic features of the endometrium and intrauterine lesions [19]; this in order to further confirm the indications for the surgical treatment. Preoperative office hysteroscopy was carried out unless the patients had been referred for surgery by other specialists external to the Hospital on the basis of an already performed hysteroscopy. Only patients who, after these pre-hospitalization procedures, had any endometrial abnormalities confirmed and therefore underwent operative hysteroscopy were included in this study. Women in which a diagnosis of uterine malformations was made were excluded from the study.

All patients gave their written informed consent after a detailed explanation of the procedure. Operative hysteroscopy was performed under general anesthesia. All procedures were assisted by video camera. A rigid unipolar 26-Fr resectoscope with an outer diameter of 8.7 mm and telescope 0° (Karl Storz, Tuttlingen, Germany) and unipolar loop electrode were used. The uterine cavity was distended with 5% sorbitol-mannitol solution and irrigation pressure, flow rate and suction pressure were electronically controlled by a combined suction and irrigation pump (Storz Hamou Endomat, Neuhausen ob Eck, Germany). In all cases, the removed endometrial tissue was sent to histopathological examination which was performed by two expert pathologists with specific training and interest in gynecologic pathology. The diagnosis of endometrial polyp(s) was made/suspected by TVS and then confirmed or refused at the time of operative hysteroscopy. The size of endometrial polyp(s) was determined by the largest diameter of the lesion measured by TVS, once the diagnosis was confirmed at hysteroscopy; when the findings of TVS were doubtful in this respect, the size was determined by measuring the largest diameter of the lesion removed “en block”. The presence of multiple endometrial polyps was finally assessed at the time of operative hysteroscopy.

Women with premalignant/malignant histologic diagnoses underwent a successive hysterectomy (simple or radical according to the type and extension of the underlying lesion) and the removed organs and tissues were examined by the same pathologists. All the pathologic reports have been provided according to the WHO Classification of Tumors of Female Reproductive Organs, 2014 [20].

### Definitions

The definitions of the specific conditions of interest are the following:

- Menopausal status: Women with serum levels of FSH > 30 IU/l, aged more 45 years and who had been amenorrhoeic for at least 12 months were defined as postmenopausal;
- Systemic Hypertension and Diabetes: Women were defined hypertensive or diabetic if they were taking regular medications for the control of the disease;
- Abnormal Uterine Bleeding (AUB): Any vaginal bleeding in postmenopausal women. In premenopausal women, AUB was defined, according to the 2011 FIGO classification [21], an acute periodic heavy bleeding or an abnormal uterine bleeding that has been present for the majority of the past six months or an intermenstrual bleeding;
- Use of Tamoxifen: regular treatment for ER + breast cancer;
- Thickened endometrium at TVS: endometrial thickness (ET) was considered abnormal if was ≥ 4 mm in postmenopausal women [22]; ET was considered abnormal in premenopausal women if was 8 mm in the proliferative phase and 16 mm in the secretory phase of the cycle [23];
- Hysteroscopic findings: the thickened endometrium and the atypical aspect of the polyps were defined according to Ianieri et al. [24]. The non-mutually exclusive criteria used to define the atypical aspects of the polyps were the following: irregular surface; presence of necrotic and/or hemorragic areas; increased vessel density; vessel dilatation and distortion; shrinkage of the vessels; easy bleeding at touch.
- Proliferative disorder: this condition is mainly associated with chronic anovulatory cycles. There is abundant proliferative endometrium associated with a mild degree of disorganization characterized by dilated glands. The histological finding is a picture that is neither normal proliferative nor hyperplastic [25].

### Data collection and handling

All the clinical cards of the study women were carefully reviewed and the data of interest were collected and reported in a preconceived template. A computerized database available for the successive analyses was then constructed. Any collected information was anonymised and de-identified prior to analysis.

### Statistical analysis

Data have been reported as means ± SD or percentages. The inferential statistical analysis in the minimal hypotheses was carried out by using Student’s *t* test and Chi Square test. Odds ratios (OR) and 95% confidence intervals (CIs) have been reported. Taking into account the high number (n = 22) of the variables considered in the study and the low prevalence of premalignant/malignant lesions, a reliable multivariate analysis could not be performed, since the proper level of significance, calculated by simply applying the Bonferroni’s correction, would be 0.05/22 = 0.00227. Therefore, to perform more than one hypothesis test simultaneously, the Holm-Bonferroni closed testing procedure was followed. In this procedure, the single p-values corresponding to the minimal hypotheses have been corrected according to their specific position in the ordinal scale of the respective levels of statistical significance. The software used was the Statistical Software SPSS release 23. Significance was set at *P* < 0.05.

## Results

One thousand and twenty consecutive women were included in the study, 403 of which were premenopausal and 617 postmenopausal. The major clinical characteristics of the women are reported in Table 1. All the clinical characteristics considered (age, BMI, systemic hypertension, diabetes mellitus, AUB, use of Tamoxifen) were found to be significantly different when they were compared according to the presence or absence of menopause. Likewise the reasons for which the women required gynecologic investigation were significantly different according to the menopausal status. Indeed, premenopausal women were more frequently symptomatic than postmenopausal ones (O.R. = 3.740, 95% C.I.: 2.868–4.876, *P* < 0.0001).

**Table 1:** Major clinical characteristics of study women according to their menopausal status

	Premenopause (n = 403 women)	Postmenopause (n = 617 women)	*p* value
Age (years)	43 ± 6.85	63.6 ± 9.42	t = − 37.84 [95% C.I.: − 21.60 to − 19.59] *P* < 0.001
BMI (kg/m^2^)	27.5 ± 5.24	28.9 ± 7.08	t = − 3.40 [95% C.I.: − 2.15 to − 0.64] *P* < 0.001
< 25	266 (66%)	184 (29.82%)	χ^2^ = 129.30, [95% C.I.: 30.13–41.83] *P* < 0.0001
25–29.9	87 (21.59%)	225 (36.47%)	χ^2^ = 25.17 [95% C.I.: 9.15–20.19] *P* < 0.0001
30–39.9	46 (11.41%)	180 (29.17%)	χ^2^ = 44.54, [95% C.I.: 12.86–22.37] *P* < 0.0001
> 40	4 (0.99%)	28 (4.54%)	χ^2^ = 10.09, [95% C.I.: 1.48–5.58] *P* < 0.002
Systemic hypertension	38 (9.43%)	310 (50.24)	χ^2^ = 180.45, [95% C.I.: 35.70–45.45] *P* < 0.0001
Diabetes mellitus	12 (2.98%)	73 (11.83)	χ^2^ = 24.97, [95% C.I.: 5.68–11.91] *P* < 0.0001
AUB	233 (57.82%)	183 (29.66%)	χ^2^ = 79.96, [95% C.I.: 22.02–34.02] *P* < 0.0001
Use of Tamoxifen	2 (0.5%)	57 (9.24%)	χ^2^ = 34.12, [95% C.I.: 6.32–11.30] *P* < 0.0001
*Symptomatology*			
Present	239 (59.31%)	173 (28.04%)	χ^2^ = 98.90, [95% C.I.: 25.17–37.06] *P* < 0.0001
Absent	164 (40.69%)	444 (71.96%)	χ^2^ = 98.93, [95% C.I.: 25.17–37.06] *P* < 0.0001
*Diagnostic office hysteroscopy*			
Yes	349 (86.6%)	500 (81.04%)	χ^2^ = 5.39, [95% C.I.: 0.88–9.94] *P* < 0.05
No	54 (13.4%)	117 (18.96%)	χ^2^ = 5.39, [95% C.I.: 0.88–9.99] *P* < 0.05

BMI body mass index, AUB = Abnormal uterine bleeding. Values are shown as mean ± SD, or n (%)

t = Student’s t test; χ^2^ = Chi-square test

The final histological examination of the endometrial abnormalities detected overall premalignant/malignant lesions in 34/1020 (3.33%) of women. Complex hyperplasia with atypia and endometrial cancer were detected in 22 (2.15%) and 12 (1.17%) cases, respectively. The postmenopausal women had a significantly higher risk of having a premalignant/malignant histopathologic diagnosis than premenopausal (Table 2).

**Table 2:** Histopathologic diagnoses in the study women stratified by menopausal status

Type of lesion	Premenopausal (n = 403 women)	Postmenopausal (n = 617 women)	*p* value
Benign	399 (90.01%)	587 (95.14%)	O.R. [95% C.I.] = 5.098 [1.782–14.582], *P* < 0.005
Premalignant/malignant	4 (0.99%)	30 (4.46%)	
*Detail of benign lesions*			
Endometrial cystic-glandular atrophy	2 (0.50%)	10 (1.62%)	χ^2^ = 2.65, [95% C.I.: − 0.37 to 2.50] *P* = 0.10, NS
Endometrium with dysfunctional proliferative disorders	19 (4.71%)	3 (0.49%)	χ^2^ = 20.54, [95% C.I.: 2.30–6.77] *P* < 0.0001
Normal endometrium	51 (12.66%)	4 (0.65%)	χ^2^ = 68.82, [95% C.I.: 8.94–15.63] *P* < 0.0001
Endometrial polyp	312 (77.42%)	540 (87.52%)	χ^2^ = 18.05, [95% C.I.: 5.34–-15.03] *P* < 0.0001
Other benign lesions*	9 (2.23%)	20 (3.24%)	χ^2^ = 0.90, [95% C.I.: − 1.24 to 3.01] *P* = 0.34, NS
Simple glandular hyperplasia	6 (1.48%)	9 (1.46%)	χ^2^ = 0.001, [95% C.I.: − 1.49 to 1.87] *P* = 0.97, NS
Complex glandular hyperplasia without atypia	0	1 (0.16%)	χ^2^ = 0.63, [95% C.I.: − 0.79 to 0.90] *P* = 0.42, NS
*Detail of premalignant/malignant lesions*			
Complex endometrial hyperplasia with atypia	4 (0.99%)	18 (2.91%)	χ^2^ = 4.26, [95% C.I.: 0.05–3.67] *P* < 0.05
Endometrial cancer	0	12 (1.94%)	χ^2^ = 7.90, [95% C.I.: 0.68–3.36] *P* < 0.005

Values are shown as n (%); * submucous myoma, polyp of the cervical canal

The detail of the final histopathologic diagnoses, together with their respective prevalence rates in the study women, stratified by menopausal status, is reported in Table 2. The premenopausal women had significantly higher risk of dysfunctional proliferative disorders than postmenopausal women (O.R. = 10.23, 95% C.I.: 3.00–34.81, *P* < 0.0005) who, on the other hand, had significantly higher risk of both benign endometrial polyps (O.R. = 2.04, 95% C.I.: 1.46–2.85, *P* < 0.0001) and of premalignant/malignant lesions (complex endometrial hyperplasia with atypia and endometrial cancer) (O.R. = 5.09, 95% C.I.: 1.78–4.58, *P* < 0.005). No cases of endometrial cancer were found in the group of premenopausal women; all the twelve cases of endometrial cancer detected were found in postmenopausal women. Four of these women at initial hysteroscopic biopsy were diagnosed to have a complex endometrial hyperplasia with atypia which then resulted to be an endometrial cancer at the successive pathologic examination after hysterectomy. All of these four women underwent both TVS and diagnostic hysteroscopy. The first woman had a 10 mm diameter polyp diagnosed at TVS and confirmed as a polyp associated with atrophic endometrium both at diagnostic and operative hysteroscopy; the second woman, who at TVS had a thickened endometrium (12 mm), hysteroscopically presented an atrophic endometrium in the context of which a 25 mm polyp was recognized; this polyp was described to have a normal aspect at office hysteroscopy while presented an irregular surface at operative procedure. The third woman had a 10 mm polyp at TVS, which presented an increased vessel density in the context of an atrophic endometrium at both diagnostic and operative hysteroscopy. The fourth woman presented a thickened endometrium associated with a 29 × 18 mm polyp at TVS, diagnosed as a polyp with increased vessel density associated with atrophic endometrium at diagnostic hysteroscopy; however, at operative hysteroscopy the lesion appeared as a diffusely thickened endometrium, easily bleeding and irregular in color, thickness and vascularity. In all 4 cases the histological examination of the hysteroscopic biopsy revealed a complex endometrial hyperplasia with atypia, while the subsequent histological diagnosis after the removal of the uterus revealed an endometrioid adenocarcinoma (G1, FIGO 1B in the first two cases; G1, FIGO 1A in the remaining ones). In three of these women with an initial diagnosis of atypical complex hyperplasia and a subsequent diagnosis of endometrial cancer after hysterectomy the malignancy originated from the implantation area of the polyp, which is often very difficult to be thoroughly assessed at hysteroscopy.

The associations between the clinical characteristics of study women and the types of endometrial lesions, benign or premalignant/malignant, are reported in Table 3.

**Table 3:** Association between the clinical characteristics of study women and the types (benign and premalignant/malignant) of endometrial lesions

Clinical characteristics of study women	Benign lesions (n = 986 women)	Premalignant and malignant lesions (n = 34 women)	Univariate analysis	*P*-value closed testing	
				Rank	Significance
Age (years)	55.27 ± 13.25	61.24 ± 9.69	* t = − 2.60, [95% C.I.: − 10.47 to − 1.46], *P* < 0.01	8	*P* = 0.04
BMI (kg/m^2^)	26.89 ± 6.53	34.24 ± 10.24	* t = − 6.30, [95% C.I.: − 9.63 to − 5.06], *P* < 0.0001	1	*P* = 0.000001
< 25	442 (44.83%)	10 (29.41%)	O.R. = 0.51 [95% C.I.: 0.24–1.08], *P* = 0.08, NS	13	*P* > 0,95, NS
25–29.9	302 (30.63%)	9 (26.47%)	O.R. = 0.81 [95% C.I.: 0.37–1.76], *P* = 0.60, NS	21	*P* > 0,95, NS
30–39.9	214 (21.7%)	11 (32.35%)	O.R. = 1.72 [95% C.I.: 0.82–3.59], *P* = 0.14, NS	15	*P* > 0,95, NS
> 40	28 (2.84%)	4 (11.76%)	O.R. = 4.56 [95% C.I.: 1.50–13.82], *P* < 0.01	9	*P* = 0.081, NS
Premenopause	399 (40.47%)	4 (11.76%)	O.R. = 0.19 [95% C.I.:0.06–0.56], *P* < 0.005	7	*P* = 0.0343
Postmenopause	587 (59.53%)	30 (88.24%)	O.R. = 5.09 [95% C.I.:1.78–14.58], *P* < 0.005	5	*P* = 0.0195
Systemic hypertension	329 (33.37%)	19 (55.88%)	O.R. = 2.52 [95% C.I.:1.26–5.04], *P* < 0.01	10	*P* = 0.09, NS
Diabetes mellitus	77 (7.81%)	8 (23.53%)	O.R. = 3.63 [95% C.I.:1.59–8.29], *P* < 0.005	6	*P* = 0.0294
Use of tamoxifen	59 (5.98%)	0 (0%)	O.R. = 0.22 [95% C.I.: 0.01–3.73], *P* = 0.29, NS	19	*P* > 0.95, NS
*AUB*					
AUB in premenopause	230/233 (98.71%)	3/233 (1.29%)	O.R. for women with AUB of having premalignant/malignant lesions: 2.20 [95% C.I.: 0.22–21.37], *P* = 0.49, NS	14	*P* > 0,95, NS
No AUB in premenopause	169/170 (99.4%)	1/170 (0.6%)			
AUB in postmenopause	163/183 (89.07%)	20/183 (10.93%)	O.R. for women with AUB of having premalignant/malignant lesions: 5.20 [95% C.I.: 2.38 – 11.35], *P* < 0.0001	2	*P* = 0.00018
No AUB in postmenopause	424/434 (97.7%)	10/434 (2.3%)			
*Transvaginal ultrasound findings*					
Women who underwent the procedure	986 (100%)	34 (100%)	O.R. = n.d		
Thickened endometrium with respect to the age or phase of the menstrual cycle	607 (61.56%)	27 (79.41%)	O.R. = 2.40 [95% C.I.:1.03–5.58], *P* < 0.05	11	*P* = 0.528, NS
Women with endometrial polyps detected at TVS and confirmed histologically	468 (47.46%)	12 (35.29%)	O.R. = 0.60 [95% C.I.:0.29–1.23], *P* = 0.16, NS	16	*P* > 0,95, NS
*Diagnostic hysteroscopy*					
Women who underwent the procedure	820 (83.16%)	29 (85.29%)	O.R. = 1.17 [95% C.I.:0.44–3.07], *P* = 0.7440	22	*P* > 0,95, NS
Endometrial polyp(s) diagnosed at hysteroscopy	758/820 (92.43%)	26/29 (89.65%)	O.R. = 0.70 [95% C.I.:0.20–2.40], *P* = 0.58, NS	20	*P* > 0,95, NS
Thickened endometrium with respect to the age or phase of the menstrual cycle	257/820 (31.34%)	12/29 (41.38%)	O.R. = 1.54 [95% C.I.:0.72–3.28], *P* = 0.25, NS	18	*P* > 0,95, NS
Multiple endometrial polyps	198/820 (24.14%)	10/29 (34.48%)	O.R. = 1.65 [95% C.I.:0.75–3.61], *P* = 0.20, NS	17	*P* > 0,95, NS
Atypical aspect of endometrial polyps	7/903 (0.78%) **	10/33 (30.3%) ***	O.R. = 55.65 [95% C.I.:19.45–159.16], *P* < 0.0001	4	*P* = 0.000396
Mean polyp size (mm)	9.27 ± 3.98	12.68 ± 7.29	* t = − 4.73, [95% C.I.: − 4.82 to − 1.99], *P* < 0.0001	3	*P* = 0.000294
Mean number of endometrial polyps/woman	1.1 ± 0.34°	1.0 ± 0.1°°	*t = -1.71, [95% C.I.: − 0.21 to 0.01], *P* = 0.08, NS	12	*P* > 0,95, NS

Values are shown either as mean ± SD or n (%); n.d. = not determined; NS = not significant;*Student’s t test; ** Value referred to the total number of polyps examined (n = 903);*** Value referred to the total number of polyps examined (n = 33); °n women = 758; °° n women = 26

Of the 986 study women with final histologic diagnosis of benign lesions, 820 (83.16%) underwent diagnostic office hysteroscopy, which documented the presence of endometrial polyp(s) in 758 (92.43%) of them. In 468 of these 758 patients (61.74%), the diagnosis of endometrial polyp(s) had already been suspected on transvaginal ultrasound, even before performing diagnostic hysteroscopy; while in the remaining 290 women (38.26%) the diagnostic suspicion had been based on the ultrasound finding of an endometrial thickening. The total number of women affected by endometrial benign lesions who presented an endometrial thickening at ultrasound was 607/986 (61.56%). The sum of the number of women with a clear ultrasound imaging of an endometrial polyp and that of women with thickened endometrium is greater than 986 (468 plus 607) because 55 women at TVS presented a thickened endometrium in the context of which an image corresponding to an endometrial polyp was not clearly identifiable.

At hysteroscopy, polyps were single in 560 women and multiple in 198 study women, with a mean of 1.1 ± 0.34 per patient.

Overall, 903 endometrial polyps with a final histologic diagnosis of benign lesions were examined, 7 of which (0.78%) presented an atypical aspect at hysteroscopy.

Moreover, 257 (31.34%) of the 820 women with final histologic diagnosis of benign lesions who underwent diagnostic office hysteroscopy presented a thickened endometrium with respect to the age or phase of the menstrual cycle.

Twenty-nine (85.29%) of the 34 study women with final histologic diagnosis of premalignant/malignant lesions, underwent diagnostic office hysteroscopy. At hysteroscopy, 12 (41.38%) of these women presented thickened endometrium with respect to the age or phase of the menstrual cycle and 26 (89.65%) of them were affected by endometrial polyps(s), which were multiple in 10 cases, with an average number of polyps of 1.0 ± 0.1 per patient.

Overall, 33 endometrial polyps with a final histologic diagnosis of premalignant/malignant lesions were examined, 10 (30.3%) of which presented an atypical aspect at hysteroscopy.

In 12 of these 26 study women (46.15%), the diagnosis of endometrial polyp(s) had already been suspected at transvaginal ultrasound, even before performing diagnostic hysteroscopy; while in the remaining 14 women (53.85%) the diagnostic suspicion had been based on the ultrasound finding of an endometrial thickening. The total number of women affected by endometrial premalignant/malignant lesions who presented an endometrial thickening at ultrasound was 27/34 (79.41%). The sum of the number of women with a clear ultrasound imaging of an endometrial polyp and that of women with thickened endometrium is greater than 34 (12 plus 27) because 5 women at TVS presented a thickened endometrium in the context of which an image corresponding to an endometrial polyp was not clearly identifiable.

At univariate analysis, the following characteristics were found to be associated with an increased risk of premalignant/malignant lesions: age, BMI, postmenopausal status, systemic hypertension, diabetes mellitus, AUB in postmenopause, thickened endometrium at TVS, mean polyp size and atypical aspect of endometrial polyp at hysteroscopy (Table 3). When the women were stratified by different ranges of BMI, the risk for premalignant/malignant lesions increased accordingly to increasing BMI, but resulted significantly higher only in the group of women with the highest BMI values (≥ 40 kg/m^2^). Conversely, neither AUB in premenopausal women nor tamoxifen use were associated with any change in the risk for premalignant/malignant endometrial lesions.

All the study women underwent TVS before surgery. The finding of a thickened endometrium at TVS was associated with an increased risk of premalignant/malignant lesions in postmenopausal women only at univariate analysis (O.R. = 2.40; 95% C.I.:1.03–5.58, *P* < 0.05). Four hundred sixty-eight (47.46%) of the 986 women with benign lesions had endometrial polyp(s) diagnosed or suspected at TVS and histologically confirmed. This rate was similar to that found in women with premalignant/malignant histology (12/34, 35.29%) (O.R. = 0.60; 95% C.I.:0.29–1.23, *P* = 0.16).

When the data reported in Table 3 were analyzed by applying the Holm-Bonferroni closed testing procedure, the most significant associations with premalignant/malignant endometrial lesions were BMI, AUB in postmenopause, overall polyp size, atypical aspect of endometrial polyps at hysteroscopy, postmenopausal status, diabetes mellitus and patient age.

## Discussion

The clinical management of the finding of an endometrial abnormality can still represent a challenge for gynecologists despite the considerable research carried out in this area. This is due to the fact that women with an endometrial abnormal finding are a very composite population which clinicians have to deal with: pre- or postmenopausal women with a large range of age, symptomatic subjects with AUB of variable extent, asymptomatic subjects in which an endometrial abnormality has been detected accidentally by an office ultrasound, patients with no, single or multiple risk factors for premalignant or malignant endometrial lesions, patients with variable surgical risk. A major clinical problem in this context is whether surgical removal should be always performed in the presence of an endometrial abnormality or it is indicated only in specific clinical settings, taking into account that the majority of endometrial lesions have a high likelihood to be benign, particularly in premenopausal women [10, 25]. The relative rarity of premalignant and malignant endometrial lesions is somehow reassuring for both clinicians and patients, when an endometrial abnormality is detected; however, this low prevalence implies the recruitment of a very high number of subjects to obtain a reliable assessment predictive of malignancy when all the potentially relevant variables—clinical, ultrasonographic and hysteroscopic—are included together in a multivariate logistic regression model. This problem has been raised in a recent, well-conducted Italian multicentric study aimed to evaluate the predictors of atypical histology in endometrial polyps removed by hysteroscopy [1]. This can also explain why many studies carried out on this subject, including the present one, are retrospective in their design [1–4, 14] or take into account only selected women with specific clinical situations, such as symptomatic or asymptomatic postmenopausal women [5, 8, 25, 26]. In this context, the present study was performed to evaluate the clinical significance, in terms of histologic endometrial atypia, of the finding of endometrial abnormalities, taking into account several risk factors for premalignant/malignant endometrial lesions which were ranked by strength of association. The results of this study, whose major limitation is its retrospective and observational design, can allow drawing some reasonable conclusions.

The stratification of the study population according to the menopausal status showed that pre-and postmenopausal women form two groups of women strongly different from each other according to all the clinical characteristic considered in the study; this difference was observed not only for age, BMI, hypertension, diabetes and use of tamoxifen, as expected, but also for the reasons of investigation, which were more often clinical, particularly AUB, in the premenopausal women and ultrasonographic in the postmenopausal ones. Indeed, in premenopausal women the presence of symptoms was more frequent than in postmenopausal ones (Table 1). In postmenopausal women, the rate of AUB was significantly lesser than that of premenopausal women (O.R. = 0.30, 95% C.I.: 0.23–0.40, *P* < 0.0001); however, in these women an endometrial abnormality detected by office ultrasound performed in asymptomatic subjects during a routinary periodic check, carried out as an extension of physical examination of the patient, was a major reason for further investigation. In our study, the rate of postmenopausal women who underwent diagnostic hysteroscopy was significantly lower than that of premenopausal patients. There are several explanations for this finding: excessive discomfort, tight stenosis of the cervix, or coexisting medical conditions preventing a safe procedure. Some patients had already undergone an office hysteroscopy at the time of their initial referral to hospital.

The stratification of the study women according to menopausal status also revealed that postmenopausal women had a significantly higher risk of having premalignant/malignant histopathologic diagnosis than premenopausal women. Indeed, in these women the overall rate of histological premalignant/malignant lesions (4.46%) was significantly higher than that found in premenopausal women (0.99%) with an O.R. of 5.09 (95% C.I.: 1.78–4.58, *P* < 0.005) (Table 2). This is in accordance with the results of other studies [3, 25] and suggests that in the vast majority of premenopausal women there is no need for an immediate surgical removal of the endometrial abnormality detected. A careful follow-up could be a reasonable management option in these patients, also taking into account that in these women only premalignant lesions and no cases of cancer were detected (Table 2). Conversely, a more aggressive attitude toward surgical removal is appropriate when endometrial abnormalities are found in postmenopausal women. A further relevant role in the management of these patients could be played by the coexistence of additional clinical characteristics associated with significantly increased risk for endometrial histological atypia. This is particularly relevant when AUB is present in postmenopausal women. Indeed, in our study premalignant/lesions were found in 10.9% of postmenopausal women with AUB and only in 2.3% of postmenopausal women without AUB (O.R. = 5.20, 95% C.I.: 2.38–11.35, *P* < 0.0001) (Table 3). All the women in which an endometrial cancer was diagnosed were postmenopausal with AUB. The low rate of premalignant/malignant lesions in postmenopausal women without AUB suggests that a conservative approach with careful surveillance could be a management option in these women.

The assessment of the association between the clinical characteristics considered in this study and the finding of premalignant/malignant endometrial lesions at histology confirmed the relevance of the known risk factors. When the P-value close testing was applied to the results obtained at univariate analysis, the most relevant clinical characteristics found to be associated with endometrial atypia or cancer were, in a decreasing order of significance, the high BMI, the presence of AUB in postmenopause, the size of polyp, the atypical aspect of the endometrial polyp at hysteroscopy, the postmenopausal status and the concomitant diabetes mellitus. Again, the premenopausal status had a significant negative association with premalignant/malignant endometrial lesions at histology.

## Conclusions

The findings of this study can further aid clinicians to properly manage the patients with endometrial abnormalities by balancing the need for an aggressive management of lesions with high likelihood to be premalignant/malignant with a more conservative approach, shared with the carefully informed patient, for lesions with low probability to have premalignant/malignant histology. However, only prospective studies, carried out on very large cohorts of women taking into account all the clinical, ultrasonographic and hysteroscopic characteristics of patients in relation to endometrial histology, will definitely clarify the overall clinical significance of the finding of an endometrial abnormality.

## Data Availability

The datasets used and/or analyzed during the current study are available from the corresponding author on reasonable request.
